# Combining nuclear genome, chloroplast fragments and morphology to solve the species delimitation of *Aquilegia incurvata* (Ranunculaceae)

**DOI:** 10.3897/phytokeys.274.161927

**Published:** 2026-05-08

**Authors:** Fei Gao, Jiao-Jie Li, Cheng Xue, Fang-Dong Geng, Lei Huang, Jian-Qiang Zhang, Xiao-Hui Zhang, Yi Ren, Ju-Qing Kang

**Affiliations:** 1 College of Life Sciences, Shaanxi Normal University, Xi’an 710119, China College of Biology, Food and Chemistry, Shaanxi Xueqian Normal University Xi’an China; 2 Key Laboratory of Medicinal Plant Resource and Natural Pharmaceutical Chemistry of the Ministry of Education, College of Life Science, Shaanxi Normal University, Xi’an 710119, China College of Life Sciences, Shaanxi Normal University Xi’an China; 3 College of Biology, Food and Chemistry, Shaanxi Xueqian Normal University, Xi’an 710100, China Key Laboratory of Medicinal Plant Resource and Natural Pharmaceutical Chemistry of the Ministry of Education, College of Life Science, Shaanxi Normal University Xi’an China

**Keywords:** *Aquilegia incurvata*, holotype and paratypes, molecular phylogenetics, morphological trait, species delimitation

## Abstract

Hybridization can be widespread among closely related plant species, and the resulting intermediate traits may complicate species delimitation and identification. Such taxonomic uncertainty can lead to misinformed conservation actions or poor resource management. *Aquilegia incurvata* is a Chinese endemic species restricted to the Qinba Mountains. Despite numerous field surveys in its reported distribution area, over a five-year period, no specimens fitting its description in “Flora Tsinlingensis” have been found, casting doubts on the validity of this species. To determine whether *A. incurvata* truly exists, we examined the type specimens of *A. incurvata* (including holotype and paratypes), as well as fresh material from four populations of *Aquilegia* occurring at the type locality. We also conducted morphological and genetic analyses to explore species boundaries between *A. incurvata* and four closely related species (*A. ecalcarata*, *A. kansuensis*, *A. yabeana* and *A. yangii*). Additionally, we integrated morphological and genetic data from 12 previously reported populations of these four closely related species. We performed morphological clustering via principal component analysis (PCA), as well as phylogenetic and population genetic analyses. Eleven floral traits from six type specimens and four populations were quantified for interspecific differentiation. Strict filtering of whole-genome resequencing data yielded 9,073 high-quality SNPs and 20 cpDNA loci from *A. incurvata*’s type specimens and its closely related species. Our study revealed two key findings: (1) The type specimens of *A. incurvata* are hybrids between *A. kansuensis*, *A. yangii* and *A. ecalcarata*, with genetic components of all three species and floral morphology intermediate between *A. kansuensis* and *A. yangii*; (2) Samples from the *locus classicus* and the provenances of the paratype specimens of *A. incurvata* cluster into two groups—One corresponds to *A. yabeana* and the other one to *A. kansuensis*. Our study confirms *A. incurvata* is not an independent species but a local hybrid.

*This paper is dedicated to the memory of Prof. Yi Ren (1959–2019), who passed away shortly before the submission of this manuscript. We will always remember him for his optimism, bravery and wisdom*.

## Introduction

Hybridization is widespread in plants, presenting substantial challenges for species delimitation (Naciri and Linder 2015; Hong 2016; Niu et al. 2018). For instance, hybridization can cause numerous continuous traits to overlap between species, blurring the boundaries between them. Additionally, a random collection of specimens may sometimes represent only the extremes within these continuous traits, or some traits can be hardly distinguishable and controversial, which leads to confusion in the recognition of distinct taxa. Fortunately, advances in sequencing technologies have facilitated the fast production of abundant molecular data, facilitating the use of neutral genetic markers to answer questions around species delimitation. In particular, species concepts such as the “integrative species concept” and “gen-morph species concept” have been proposed, providing a theoretical framework for comprehensive verification of species relationships using multiple types of data (e.g., morphological, molecular and geographical) (Hong 2016, 2020; Liu 2016; Niu et al. 2018).

It has been known for decades that hybridization in *Aquilegia* is common (Grant 1952). Previous studies have demonstrated that postzygotic reproductive isolation among species in *Aquilegia* is weak, promoting frequent hybridization (Prażmo 1961; Groh and Cronk 2020). For example, Groh and Cronk (2020) have reported hybrid individuals with flower color variation in sympatric populations of the North American taxa *A. flavescens* and *A. formosa* (Groh and Cronk 2020). Hybridization has also been reported between Chinese species of *Aquilegia*, where frequent gene flow indicates that reproductive isolation has not yet been fully established (Geng et al. 2024; Liu et al. 2025).

The Chinese endemic *Aquilegia incurvata* P.K.Hsiao was first described in “Flora Tsinlingensis” by Hsiao in 1974 (Instituto Botanico Boreali-Occidentali 1974). This purple-flowered species is restricted to the Qinba Mountains (comprising the Qinling Mountains and Daba Mountains) at altitudes of 1000–2800 m. This area is considered a biodiversity hotspot (Myers et al. 2000). The holotype for this species (i.e. WUK 0122218) was collected from Ningshan County, Shaanxi Province, located in the Qinba Mountains. In recent years, this area has become a research hotspot for Aquilegia (e.g. Xue et al. 2020; Xue et al. 2021; Huang et al. 2022; Geng et al. 2022; Liu et al. 2025), but few studies have focused on *A. incurvata*. Over the years, the occurrence of at least four other *Aquilegia* species with similar purple flowers has been confirmed in the Qinba Mountains (Instituto Botanico Boreali-Occidentali 1974; Luo et al. 2018): *A. ecalcarata* Maxim., *A. yabeana* Kitag., *A. kansuensis* (Brühl) Erst and *A. yangii* Luo & Li. Field surveys conducted over a five-year period, however, revealed no population of *Aquilegia* fully matching the morphological description of *A. incurvata*. Additionally, examination of morphological descriptions of *A. incurvata* and its four closely related species in the “Flora of China” indicates that multiple floral traits of these species show numerical overlap and continuity, which makes delimitation and taxonomic identification of these species difficult (Suppl. material 1: table SS1). For example, the sepal length of *A. incurvata* overlaps with *A. kansuensis*, *A. yangii*, *A. yabeana* and *A. ecalcarata*, the spur length of *A. incurvata* overlaps with *A. kansuensis*, and the length of its follicles overlaps with *A. kansuensis* and *A. yabeana* (Suppl. material 1: table SS1).

Given the absence of natural populations in the wild conforming to the morphological description of *A. incurvata*, coupled with weak reproductive isolation between different species in *Aquilegia*, as well as the sympatric distribution of multiple species in the Qinba Mountains (Wang et al. 2012; Xu et al. 2019), we hypothesized that *A. incurvata* might be a hybrid taxon. To test this hypothesis, we employed an integrative approach combining nuclear genomic data, chloroplast loci and morphological data to investigate species delimitation of *A. incurvata*. Our aims were: (1) to determine whether genetics and morphological evidence support *A. incurvata* as an independent species; (2) to clarify the taxonomic identity of the type specimens (including holotype and paratypes) and fresh specimens collected from the type locality.

## Materials and methods

### Taxon sampling

We undertook a detailed morphological study of the holotype specimen of *A. incurvata* and eight paratypes (Table 1; Suppl. material 1: fig. S1). Floral traits were measured for the holotype (S_Typus) and five paratypes (S_01, S_04, S_05, S_06 and S_07), for which floral trait data were available and sufficient. Concurrently, with permission from Northwest A&F University herbarium (WUK), we removed small leaf fragments from the holotype (S_Typus) and two paratypes (S_01 and S_02) for genetic analysis, ensuring there was no compromise to the specimens’ integrity or future studies.

**Table 1.** Collection details for the type material of *A. incurvata* included in this study at the herbarium CODE.

No.	Collection information	Elevation/m	Collection Date	Type Specimens Status	Voucher	Genome sequence	Traits measurement
S_Typus	Guankou, Ningshan, Shaanxi, China	1050	May 14, 1959	Holotype	WUK 0122218	√	√
S_01	Dongan, Chengkou, Sichuan, China	1250	July 2, 1959	Paratype	WUK 0423362	√	√
S_02	Taohe, Ankang, Shaanxi, China	1920	July 29, 1959	Paratype	WUK 0423366	√	–
S_03	Sijihe, Langao, Shaanxi, China	1950	July 18, 1959	Paratype	WUK 0133927	–	–
S_04	Huangguan, Shiquan, Shaanxi, China	1020	June 19, 1959	Paratype	WUK 0123903	–	√
S_05	Jiangkou, Shiquan, Shaanxi, China	1700	June 13, 1959	Paratype	WUK 0122785	–	√
S_06	Taohe, Ankang, Shaanxi, China	1800	July 22, 1959	Paratype	WUK 0137389	–	√
S_07	Longgangwan, Chengkou, Sichuan, China	1900	July 16, 1959	Paratype	WUK 0137370	–	√
S_08	Gaojiaba, Foping, Shaanxi, China	1300	July 14, 1952	Paratype	WUK 0059584	–	–

**Figure 1.**
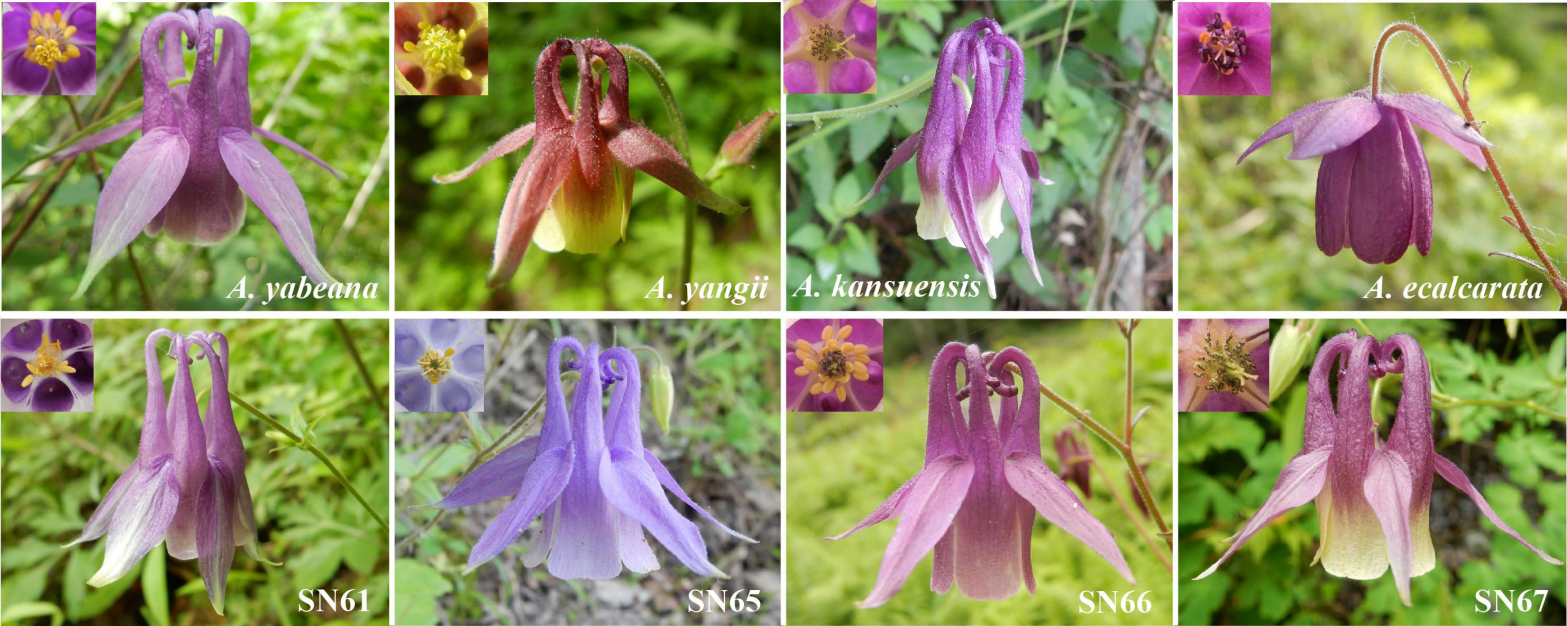
The floral and androecium morphology of *A. yabeana*, *A. yangii*, *A. kansuensis* and *A. ecalcarata*, and samples from four populations (SN61, SN65, SN66 and SN67) found at the Qinba Mountains, where the holotype (S_Typus) and paratypes of *A. incurvata* were collected in 2018.

**Figure 2.**
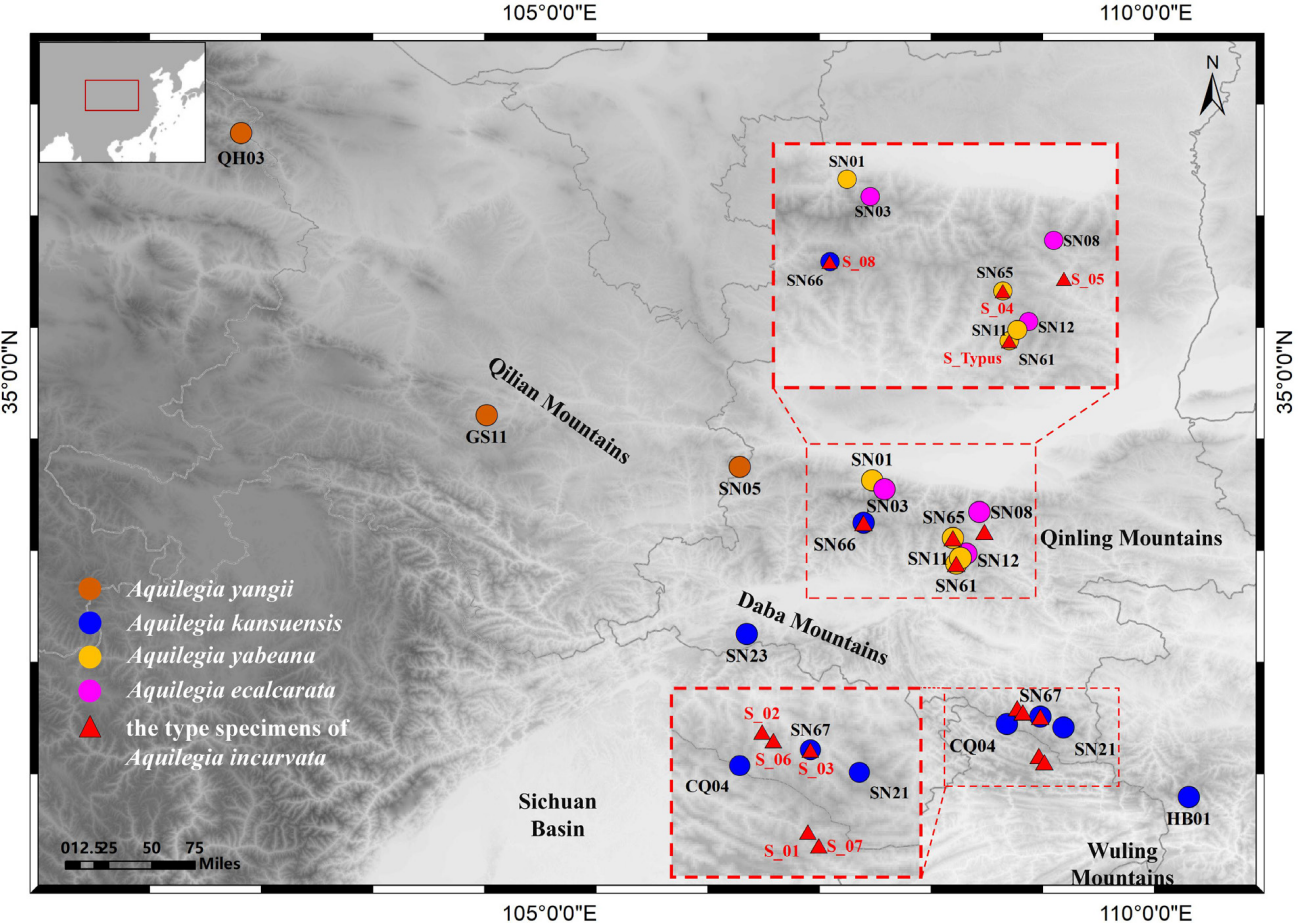
Distribution of the type specimens of *A. incurvata* and populations of its closely related species *A. yabeana*, *A. yangii*, *A. kansuensis* and *A. ecalcarata*. The labels in black font represent extant populations, and those in red font indicate the type specimens of *A. incurvata*. The topographic map is from http://srtm.csi.cgiar.org/.

In 2018, we conducted field trips to the localities from where the holotype and paratypes of *A. incurvata* originated and collected fresh samples for morphological and genetic analyses from four populations (SN61, SN65, SN66 and SN67) (Tables 2, 3; Figs 1, 2). Five to fifteen flowers were collected per population (50 total) and fixed in FAA solution (70% alcohol: formalin: acetic acid = 90: 5: 5 by volume) for later examination. To ensure a comprehensive sampling of the four populations, we collected samples at the same altitudes recorded for the type specimens and across an extended altitudinal range. Based on morphological traits and description in “Flora of China”, plants from populations SN61 and SN65 were identified as *A. yabeana* and samples from the populations SN66 and SN67 as *A. kansuensis* (Fig. 1).

**Table 2.** Sample information for 16 populations of *Aquilegia yabeana*, *A. yangii*, *A. kansuensis* and *A. ecalcarata* included in this study.

No.	Population(s)	Taxon	Locality	Latitude, Longitude	Altitude (m)	Number of individuals
1	SN61	*A. yabeana*	Ningshan County, Shaanxi	33°23'18"N, 108°24'46"E	1934*	3
2	SN65	*A. yabeana*	Ningshan County, Shaanxi	33°36'14"N, 108°23'4"E	1356*	5
3	SN01	*A. yabeana*	Mei County, Shaanxi	34°5'16"N, 107°42'28"E	1182	5
4	SN11	*A. yabeana*	Ningshan County, Shaanxi	33°26'6"N, 108°26'49"E	1582	5
5	QH03	*A. yangii*	Tu Autonomous County of Huzhu, Qinghai	36°55'8"N, 102°24'57"E	2517	5
6	SN05	*A. yangii*	Feng County, Shaanxi	34°12'0"N, 106°35'45"E	1578	5
7	GS11	*A. yangii*	Zhang County, Gansu	34°38'2"N, 104°28'26"E	2451	5
8	SN23	*A. kansuensis*	Nanzheng District, Shaanxi	32°48'0"N, 106°39'16"E	1562	5
9	HB01	*A. kansuensis*	Xingshan County, Hubei	31°26'2"N, 110°21'46"E	1608	5
12	CQ04	*A. kansuensis*	Chengkou County, Chongqing	32°2'34"N, 108°50'9"E	2245	5
11	SN21	*A. kansuensis*	Zhenping County, Shaanxi	32°1'1"N, 109°18'36"E	2079	5
10	SN66	*A. kansuensis*	Taibai County, Shaanxi	33°43'54"N, 107°28'6"E	1131*	5
13	SN67	*A. kansuensis*	Langao County, Shaanxi	32°06'22"N, 108°51'37"E	1630*	5
14	SN03	*A. ecalcarata*	Mei County, Shaanxi	34°0'46"N, 107°48'32"E	2770	5
15	SN08	*A. ecalcarata*	Hu County, Shaanxi	33°49'22"N, 108°36'18"E	2439	5
16	SN12	*A. ecalcarata*	Ningshan County, Shaanxi	33°28'18"N, 108°29'46"E	2167	5

**Table 3.** Principal component analysis (PCA) for the floral traits.

Eigen value	6.775	2.106	1.227
Proportional variation (%)	61.589	19.148	11.159
Cumulative variation (%)	61.589	80.736	91.895
**Traits**	**PC1**	**PC2**	**PC3**
Petal blade width	**0.949**	0.12	0.198
Sepal length	**0.941**	0.237	0.097
Petal length	**0.93**	0.325	0.039
Sepal width	**0.922**	-0.27	0.15
Petal blade length	**0.888**	-0.162	-0.281
Spur aperture	**0.843**	0.408	0.231
Spur arc length	**0.825**	**0.543**	0.09
Petal blade length/width	**-0.537**	**-0.501**	**-0.528**
Sepal length/width	-0.099	**0.944**	-0.141
Petal length/spur length	-0.288	**-0.809**	-0.31
Color of dehiscent anthers	-0.002	0.006	**0.951**

The bold values are the highest absolute values among 4 principal components (PCs) for each.

To clarify the species delimitation of *A. incurvata* and its relatives, we also incorporated samples from another 12 populations of the four closely related species (Figs 1, 2; Table 2), whose distributions overlap with the documented distribution of *A. incurvata*. Floral morphology and genomic data for these 12 populations, along with genomic data from the four populations sampled in 2018 (SN61, SN65, SN66 and SN67), were sourced from Xue et al. (2020) and our own contributions (Geng et al. 2022).

### Genome sequencing, assembly and gene annotation

DNA extraction method and genome skimming approach followed Zeng et al. (2018). This generated approximately 8 Gb of sequencing data per sample with an average depth of 8×. Raw data processing adhered to the pipeline reported in Geng et al. (2022). For populations SN61, SN65, SN66 and SN67, single-nucleotide polymorphisms (SNPs) were called from raw genomic data using the bioinformatics pipeline described in Geng et al. (2022).

For chloroplast DNA data, we used *A. ecalcarata* (GenBank accession: MK569475) (Zhai et al. 2019) as the reference to assemble sequences, with gaps repaired using MITObim v1.9 (Hahn et al. 2013). Annotation was performed in Geneious 10.2.2 (http://www.geneious.com/). Due to incomplete genetic information from type specimens, complete plastid genome data could not be assembled; thus, we screened for fragments that could yield full-length sequences. Twenty chloroplast DNA fragments (i.e., *psbE-petL*, *psaI-accD*, *ndhC-trnV*, *ndhJ-trnF*, *atpH-atpI*, *rps16-trnQ*, *trnK-rps16*, *ndhA intron*, *rpl32-trnL*, *trnS-trnG*, *trnC-rpoB*, *trnD-psbM*, *ycf6-psbM*, *trnC-ycf6*, *trnD-trnT*, *trnS-trnG*, *rps4-trnF*, *rps12-rpl20*, *psbB-psbH*, and *matK*) (Fior et al. 2013) were obtained by sequence alignment in Geneious 10.2.2. Sequence alignments were conducted in MAFFT 7.3 using default parameters (Katoh and Standley 2013).

For the nuclear genome sequence data of all samples, we used *A. oxysepala* var. kansuensis as a reference (Xie et al. 2020). This species has been identified as *A. yangii* by Luo et al. (2018), with a genome size of 293.21 Mb and seven chromosomes. Clean reads were then mapped to this reference genome using BWA-MEM v.0.7.15 with default parameters (Li and Durbin 2009). After alignment, mapped reads were sorted and duplicates removed using SAMtools v.1.3.1 (Li 2011), with SNPs called and converted to the Variant Call Format. Analyzing the SNPs filtered with different parameter combinations, we determined that only the combination ‘--maf 0.01 --max-meanDP 30 --minDP 5 --minQ 25 --max-missing 1 --remove-indels’ (where “maf” denotes minor allele frequency) yielded SNPs that constructed the most robust phylogenetic tree with the highest support values at branch nodes. Due to incomplete genetic data generated from type specimens, long contiguous and complete sequences were not obtained. Finally, a total of 9,073 SNPs were retained from the initial 16,200,122 SNPs.

### Phylogenetic and population genetic analyses

Three genetic datasets were used in this study, as follows:

1. **Multi-locus cpDNA dataset of *Aquilegia*** , including 20 highly variable chloroplast loci (*psbE-petL*, *psaI-accD*, *ndhC-trnV*, *ndhJ-trnF*, *atpH-atpI*, *rps16-trnQ*, *trnK-rps16*, *ndhA intron*, *rpl32-trnL*, *trnS-trnG*, *trnC-rpoB*, *trnD-psbM*, *ycf6-psbM*, *trnC-ycf6*, *trnD-trnT*, *trnS-trnG*, *rps4-trnF*, *rps12-rpl20*, *psbB-psbH* and *matK*) (Fior et al. 2013) from 82 individuals of *Aquilegia* genus and *Semiaquilegia adoxoides* (DC.) Makino. These individuals included the holotype (S_Typus) of *A. incurvata*, a representative individual of *A. kansuensis* (CQ0428), as well as 80 individuals of *Aquilegia* species used in Fior et al. (2013) and outgroup *S. adoxoides*. The dataset was constructed to resolve the phylogenetic relationship of *A. incurvata* within *Aquilegia*. Given that cpDNA is maternally inherited (Jeffrey et al. 2018), haploid and non-recombined, these sequences were concatenated for all individuals before analyses.
2. **Multi-locus cpDNA dataset of***A. incurvata***and its relatives**, including 20 highly variable chloroplast loci of the holotype (S_Typus) of *A. incurvata* and 79 individuals from its relatives (Table 2), which included six populations of *A. kansuensis*, three populations of *A. yangii*, four populations of *A. yabeana* and three populations of *A. ecalcarata*, as well as the published plastid genome of *A. coerulea* (GenBank accession: MK569474), which was used as the outgroup. Because the data quality of the chloroplast locus of two *A. incurvata* specimens were poor (i.e. S_01 and S_02), their data were not included in this data set.
3. **Genome-wide SNPs dataset**, including 9,073 genome-wide SNPs from three type specimens (S_Typus, S_01 and S_02) of *A. incurvata* and 79 individuals of its relatives, which included six populations of *A. kansuensis*, three populations of *A. yangii*, four populations of *A. yabeana*, three populations of *A. ecalcarata* and *A. oxysepala* (JL01), which was used as the outgroup.

Phylogenetic consensus trees of the two multi-locus cpDNA and the genome-wide SNPs dataset were constructed by the maximum-likelihood (ML) method and applying a Bayes Test (Anisimova et al. 2011) using IQ-TREE 1.6.8 (Nguyen et al. 2015). ML analyses employed 50,000 replicates with the SH-like approximate likelihood ratio test (SH-aLRT) (Guindon et al. 2010). Based on the Bayesian Information Criterion (BIC) score calculated by ModelFinder (Kalyaanamoorthy et al. 2017) in PhyloSuite v1.1.16 (Zhang et al. 2020), the best-fit models for the three datasets were revealed: TVM+R3+F (multi-locus cpDNA dataset of *Aquilegia*), F81+I+F (multi-locus cpDNA dataset of *A. incurvata* and its relatives) and TVM+R3+F (Genome-wide SNPs dataset).

For population genetic inference, we used Plink v1.07 (Purcell et al. 2007) to generate input files for ADMIXTURE (Alexander et al. 2009). The species complex structure was investigated in ADMIXTURE with four independent runs per *K* value (*K* = 2-10). Log likelihood values were used to assess convergence across runs, retaining the run with the lowest log likelihood. The optimal number of genetic clusters (K) was determined by selecting the value with the lowest cross-validation (CV) error, as this best describes the population structure (Alexander et al. 2009). Barplots of the genetic assignments of the different individuals were illustrated using an R script (v3.6.3).

### Morphological measurement and analysis

To explore the morphological variation between *A. incurvata* and its relatives, floral traits were measured from six type specimens (S_Typus, S_01, S_04, S_05, S_06 and S_07) of *A. incurvata*, two populations of *A. yabeana* (SN61 and SN65, n = 5 and 15) and two of *A. kansuensis* (SN66 and SN67, n = 15 and 15), which were collected from the locality where the holotype (S_Typus) and paratypes of *A. incurvata* were gathered, respectively. A total of 11 floral traits were measured per sample, including ten quantitative traits: sepal length, sepal width, sepal length/width ratio, petal length, petal length/spur length ratio, petal blade length, petal blade width, petal blade length/width ratio, spur arc length and spur aperture; one qualitative trait: color of dehiscent anthers (Suppl. material 1: table SS2) (Data S1). Floral trait measurements followed the protocol described in our previous study (Xue et al. 2020). Concurrently, we retrieved the data of the 11 floral traits of other *Aquilegia* species used in this study (Xue et al. 2020).

Before analyses, Z-score was used to normalize the data and a principal component analysis (PCA) was conducted using SPSS software (SPSS v22.0 for Windows, SPSS Inc., Chicago, IL, USA). The 3D-PCA plot and the violin plots were generated using 3D Scatter tools and Violin tools in Hiplot (https://hiplot.com.cn).

## Result

### Genome resequencing and chloroplast loci features

The combined length of 20 chloroplast loci from the holotype (S_Typus) of *A. incurvata* was 20,602 bp (GC content: 33.8%). In the multi-locus cpDNA dataset of *Aquilegia* samples, sequence lengths ranged from 18,136 to 20,602 bp (GC content: 33.6–33.9%), and the length of the aligned dataset was 21,254 bp. For the 20 loci cpDNA dataset of *A. incurvata* and its relatives, the sequence lengths ranged from 20,272 to 20,602 bp (GC content: 33.7–33.8%) and the aligned matrix measured 20,421 bp. From an initial set of 16,200,122 single-nucleotide polymorphisms (SNPs) generated via whole-genome resequencing, a total of 9,073 high-quality SNPs were retained after stringent filtering.

### Phylogenetic relationships of cpDNA datasets

To resolve the phylogenetic position of *A. incurvata* within the genus *Aquilegia*, we reconstructed the phylogenetic tree using the multi-locus cpDNA dataset (Suppl. material 1: fig. S2). The topology of the inferred tree was consistent with the phylogenetic tree reported by Fior et al. (2013). The holotype (S_Typus) of *A. incurvata* clustered within a monophyletic group alongside five species endemic to China (*A. yabeana*, *A. yangii*, *A. kansuensis*, *A. ecalcarata*, and *A. rockii*). Four out of these five species (excluding *A. rockii*) exhibited overlapping distributions with *A. incurvata* in Shaanxi Province, Chongqing City and Gansu Province, belonging to the Qinba Mountains.

To further resolve the phylogenetic relationship of *A. incurvata* and its sympatric relatives, we inferred a phylogenetic tree using the 20 loci cpDNA dataset comprising the type specimen of *A. incurvata* and 78 individuals from its four relatives, employing ML/aBayes methods (Suppl. material 1: fig. S3). Phylogenetic analysis revealed that *A. incurvata* and its relatives diverged into two clades. One clade consisted of *A. yangii* and a single *A. kansuensis* individual (SN66-18), mainly distributed in the Qilian Mountains. The remaining individuals, including the type specimen of *A. incurvata*, formed the other clade, mainly distributed in the Qinba Mountains and the northern mountainous region of the Wuling Mountains. None of the five species formed a monophyletic group. The chloroplast haplotypes of *A. incurvata* haplotype (S_Typus) and some individuals of *A. kansuensis* (SN67, SN23 and HB01) and *A. ecalcarata* (SN08) clustered into a strongly supported monophyletic clade.

### Phylogenetic relationships and population structure of Genome-wide SNP datasets

**Figure 3.**
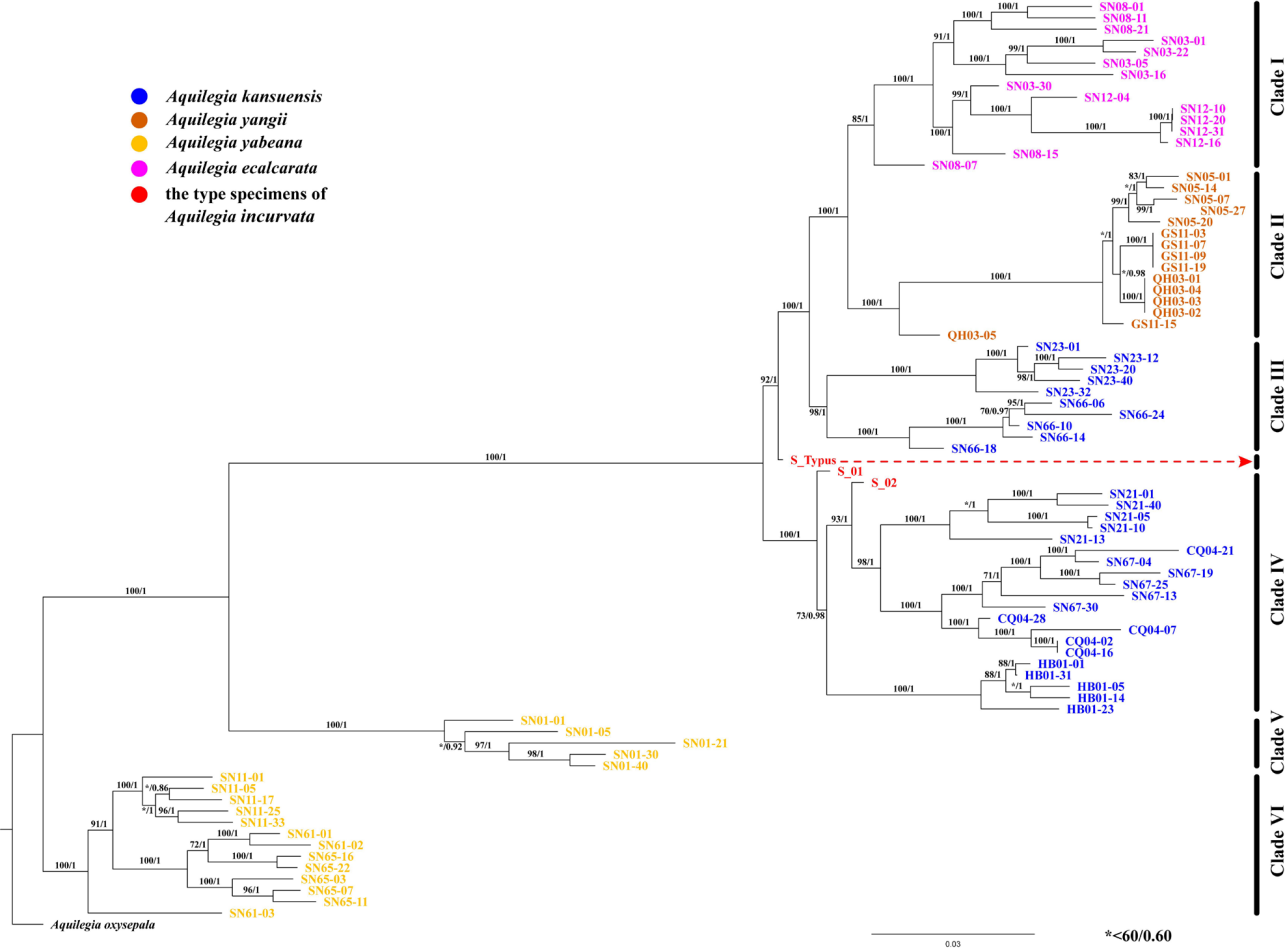
Phylogenetic relationships of *A. incurvata* and its closer relatives *A. yabeana*, *A. yangii*, *A. kansuensis* and *A. ecalcarata* based on a genome-wide SNPs dataset. The numbers by the branches represent ML bootstrap values / aBayes values.

The ML/aBayes phylogenetic tree inferred from the 9,073 SNPs dataset of the nuclear genome showed that the type specimens of *A. incurvata* and its relatives diverged into six clades and one independent branch containing only the *A. incurvata* holotype (S_Typus) (Fig. 3). Clade I and Clade II each included all of the individuals of *A. ecalcarata* and *A. yangii*, respectively. Clade III included two populations of *A. kansuensis* (SN23 and SN66). Clade IV included two paratypes of *A. incurvata* (S_01 and S_02) and four additional populations of *A. kansuensis*. Clade V and Clade VI included all populations of *A. yabeana*. In the phylogeny, S_Typus was located between Clade III and Clade IV (Fig. 3).

**Figure 4.**
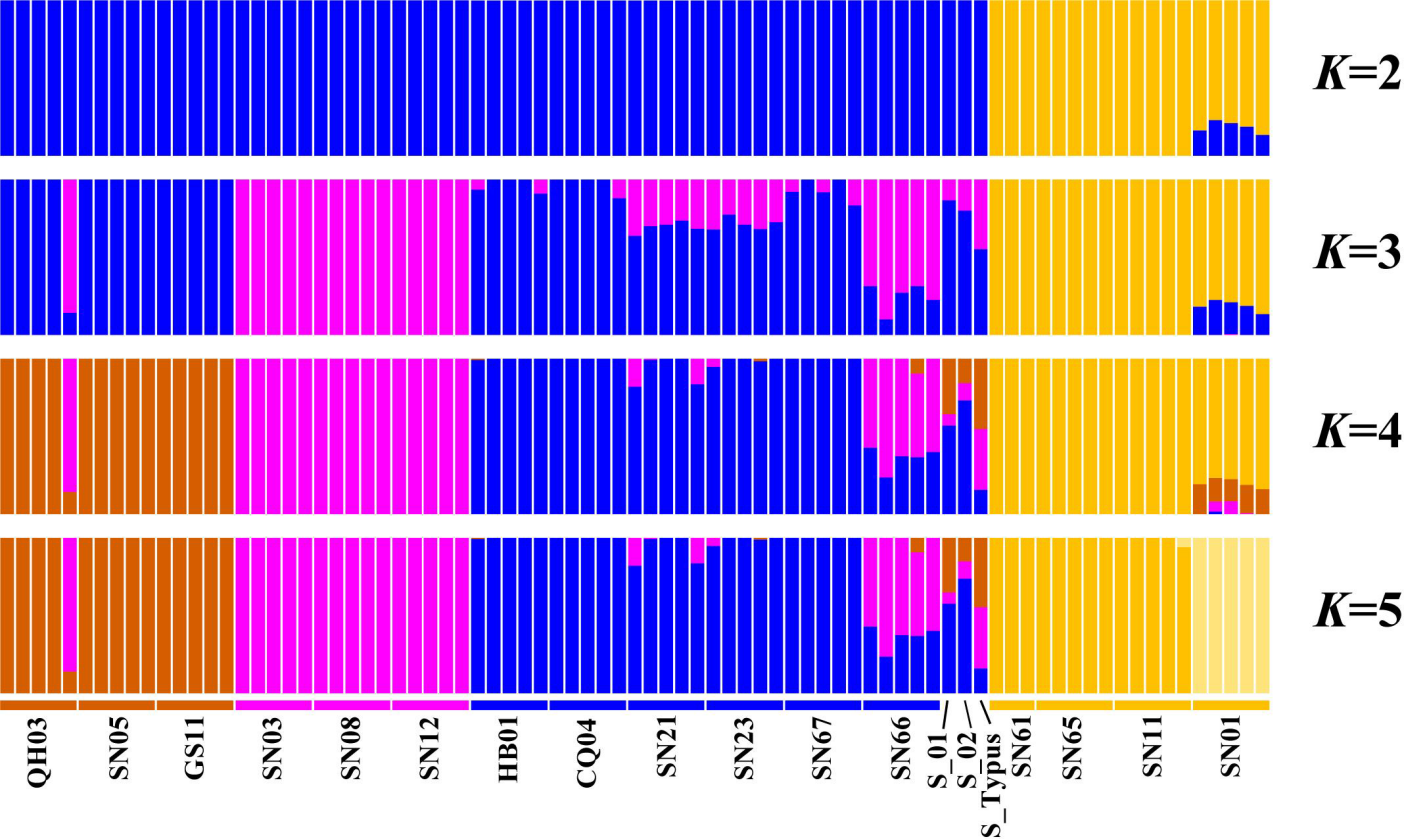
Population genetic structure analysis of *A. incurvata* type material and affin species *A. yabeana*, *A. yangii*, *A. kansuensis* and *A. ecalcarata* at *K* = 2–5 on a genome-wide SNPs dataset. The optimal *K* value is 4. Each vertical bar represents an individual.

ADMIXTURE cross-validation errors (CV) suggested that *K* = 4 had the lowest CV error, designating it as the optimal *K* value (Suppl. material 1: fig. S4). At *K* = 2, all populations of *A. yabeana* clustered into a single group, whose genetic composition was dominated by the genetic component represented by the yellow-colored segment in the ADMIXTURE plot (Fig. 4); this group corresponded to Clade V and Clade VI in Fig. 3. All other samples formed a separate cluster with a distinct genetic component. At *K* = 3, *A. ecalcarata* formed an independent cluster, whose primary genetic component was indicated by the purple-colored segment in the plot and corresponded to Clade I in Fig. 3. Meanwhile, the three type specimens (S_Typus, S_01 and S_02) of *A. incurvata* exhibited a genetic composition consisting of the two components marked by purple and blue segments in the plot—this profile was similar to the genetic makeup of most individuals of *A. kansuensis* (Fig. 4). When *K* = 4 (the optimal value), a new genetic component (derived from the one previously marked by the blue segment) emerged, which was visualized as the brown-coloured segment in the plot (Fig. 4). This brown-marked component corresponded to Clade II in Fig. 3 and represented the unique genetic signature of *A. yangii* (except for one individual from QH03, whose genetic composition contained a large proportion of the component characteristic of *A. ecalcarata*). At this optimal *K* value, the four relatives of *A. incurvata* (*A. kansuensis*, *A. yangii*, *A. yabeana* and *A. ecalcarata*) were fully distinguished by their distinct genetic component profiles (Fig. 4). However, the genetic composition of the *A. incurvata* type specimens was similar to that of one population (SN66) of *A. kansuensis*, with both containing three genetic components (shown as purple, blue and brown segments in the plot). At *K* = 5, only intraspecific genetic structure increased—specifically, the *A. yabeana* population (SN01) separated into a distinct cluster (Fig. 4). Higher *K* values thereafter generated redundant clusters that explained minimal additional genetic variation.

### Statistical analyses of the morphological dataset

The most informative principal components (PCs) with eigenvalue > 1.0 and the vector loadings of 11 floral traits of *A. incurvata* and its relatives explained this percentage change (Table 3). The first three PCs cumulatively explained 91.895% of the total variation. PC1 (61.589% of variation) was mainly comprised of petal blade width, sepal length, petal length, sepal width, petal blade length, spur aperture, spur arc length and petal blade length/width. PC2 (19.148% of variation) was mainly comprised of spur arc length, petal blade length/width, sepal length/width, and petal length/spur length. PC3 (11.159% of variation) was mainly composed of the color of dehiscent anthers. PC1 was interpreted as reflecting floral size; PC2 was floral morphological proportions; PC3 was the color of dehiscent anthers.

**Figure 5.**
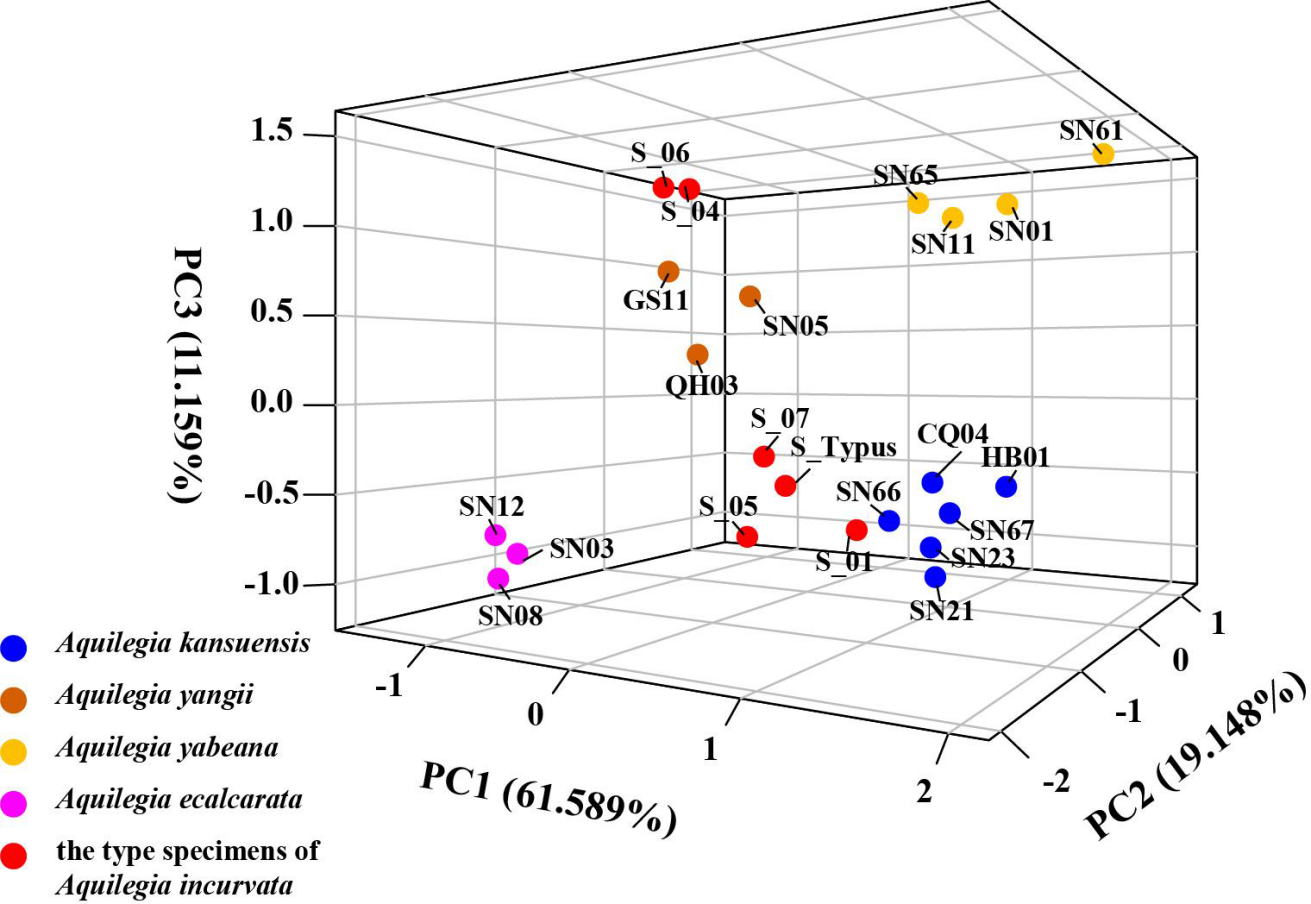
3D-PCA plot of Principal Component Analysis (PCA) results based on the morphological measurements of 22 individuals representing *A. incurvata* and related species *A. yabeana*, *A. yangii*, *A. kansuensis* and *A. ecalcarata*.

**Figure 6.**
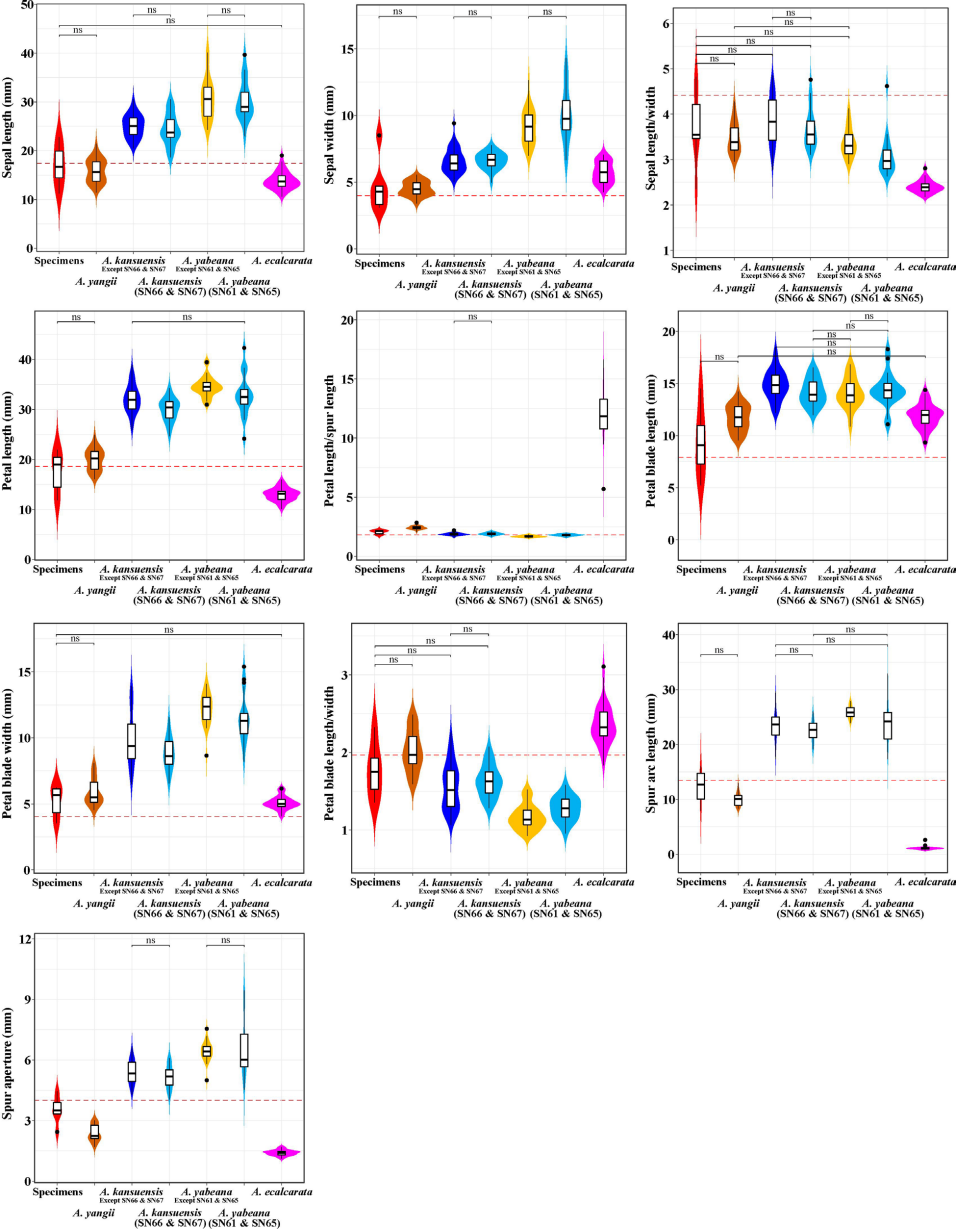
Violin plots of ten quantitative traits of *A. incurvata* and related species *A. yabeana*, *A. yangii*, *A. kansuensis* and *A. ecalcarata*. ‘ns’ means no significant difference.

**Figure 7.**
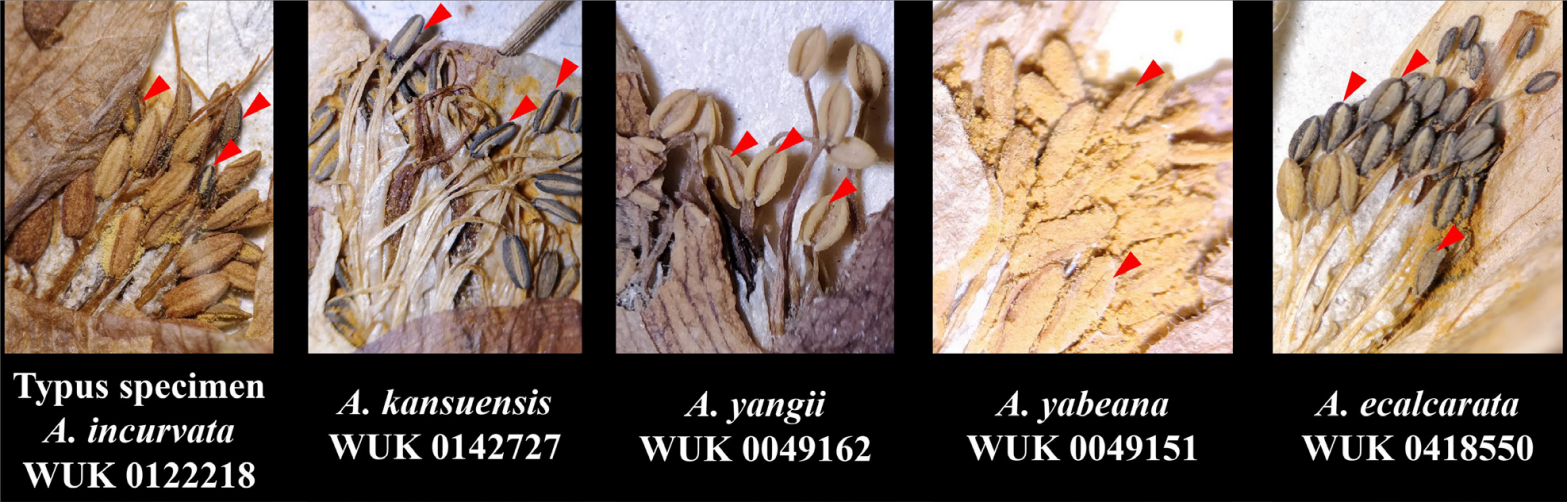
Anther colour in the type of *A. incurvata* and related species *A. yabeana*, *A. yangii*, *A. kansuensis* and *A. ecalcarata* observed from specimens at the Plant Herbarium of Northwest Agriculture and Forestry University (NWFC). The specimens of *A. yabeana*, *A. yangii*, *A. kansuensis* and *A. ecalcarata* were all collected in the same year or at least 20 years before the holotype of *A. incurvata*. The color of the dehiscent anthers of these observed specimens was consistent with the field observations, indicating that the color of the dehiscent anthers of the herbarium specimens was not affected by the year. The color of the dehiscent anthers of the holotype of *A. incurvata* is black.

The 3D-PCA plot, excluding the type specimens of *A. incurvata*, revealed that *A. kansuensis*, *A. yangii*, *A. yabeana* and *A. ecalcarata* were clustered into four groups, respectively, each forming a separate group with notable floral trait differences (Fig. 5). However, the six type specimens (S_Typus, S_01, S_04, S_05, S_06 and S_07) of *A. incurvata* did not cluster together. They were instead scattered around *A. kansuensis* and *A. yangii*, linking the two species to form a large group. Furthermore, the holotype (S_Typus) of *A. incurvata* showed greater similarity to *A. kansuensis*. When compared to *A. kansuensis* and *A. yangii*, the flower size of the holotype was between the two species on PC1; on PC2, its floral morphological proportions did not differ substantially from those of *A. kansuensis*, *A. yangii* and *A. yabeana*; and on PC3, the holotype was the most similar to *A. kansuensis* (Fig. 5). That is, the color of dehiscent anthers of *A. incurvata* was black, which was different from the yellow of *A. yangii*. Four populations (SN61, SN65, SN66 and SN67) sampled from the *locus classicus* and the provenances of the paratype specimens of *A. incurvata* in the Qinling Mountains were identified as: SN61 and SN65 belonged to *A. yabeana*, whereas SN66 and SN67 belonged to *A. kansuensis*. This classification was consistent with their phylogenetic traits and morphological results (Fig. 3 and Fig. 5). To further validate the morphological similarity between *A. incurvata* type specimens and their relatives, independent samples t-tests were conducted. The results revealed significant differences in most floral traits between the compared groups (Fig. 6). Interestingly, the group of *A. incurvata* type specimens showed no significant differences in eight quantitative traits when compared to *A. yangii*, except for the color of dehiscent anthers (Fig. 6). For two key quantitative traits (sepal length/width and petal blade length/width), no statistically significant differences were detected when the *A. incurvata* specimen group was compared against three distinct groups: the *A. kansuensis* group excluding SN66 and SN67, the *A. kansuensis* group restricted to SN66 and SN67, and the *A. ecalcarata* group (Fig. 6). In addition, dehiscent anthers were consistently black in color across all these groups (Fig. 7). For the holotype of *A. incurvata*, only petal blade length was shorter, whereas all other quantitative traits fell within or between the range of those in *A. kansuensis* (except SN66 and SN67) and *A. yangii*. Furthermore, eight quantitative traits showed no significant differences between *A. kansuensis* (SN66 and SN67) and other *A. kansuensis* populations, and the color of dehiscent anthers was black in both (Figs 1, 6). Conversely, four quantitative traits did not differ significantly between the group of *A. yabeana* (SN61 and SN65) and other *A. yabeana* populations, and the color of dehiscent anthers was yellow in both (Figs 1, 6).

## Discussion

This study utilized multiple datasets (nuclear genome data, chloroplast loci and morphological data) to investigate species delimitation of *A. incurvata*. When specimens representing sites recorded in the holotype and type specimens of *A. incurvata* were excluded, nuclear genome SNP phylogenetic analysis clearly resolved *A. kansuensis*, *A. yangii*, *A. yabeana* and *A. ecalcarata*. Moreover, PCA analysis of floral traits demonstrated pronounced morphological differences among these four related species. When including the type specimens of *A. incurvata* and the four populations (SN61, SN65, SN66 and SN67) collected from the sampling sites of *A. incurvata* specimens, genetic and morphological analysis revealed the complex genetic and morphological relationships between *A. incurvata* and its four relative species.

Given that the type specimen is the most basic and most important basis for the delimitation of a species in the taxonomic concept, the holotype and paratypes of *A. incurvata* were initially subjected to detailed study. Admixture analysis revealed that the genetic compositions of S_Typus, S_01 and S_02 remained similar and admixed across all *K* values but K = 2 (Fig. 4). Especially at the optimal *K* value of 4, these three type specimens of *A. incurvata* were each composed of the genetic compositions from *A. kansuensis*, *A. yangii* and *A. ecalcarata*. Populations of the latter three species exhibit admixed genetic compositions, and interspecific hybridization has also been reported in previous studies (Geng et al. 2024; Liu et al. 2025; Peng et al. 2025; Xue et al. 2021). In this study, it was found that the three type specimens of *A. incurvata* did not form a monophyletic group but instead exhibited a polyphyletic relationship with *A. kansuensis* in the phylogenetic analysis of nuclear genome SNPs. Meanwhile, the phylogenetic analysis of the chloroplast loci showed that the holotype of *A. incurvata* shared the closest ancestral haplotype with certain populations of *A. kansuensis* and *A. ecalcarata* (Suppl. material 1: fig. S3). Therefore, genetic evidence indicated that the type specimens of *A. incurvata* likely represent hybrid individuals among *A. kansuensis*, *A. yangii* and *A. ecalcarata*.

Furthermore, PCA analysis of floral traits showed that the quantitative floral morphological variation of holotype and paratypes of *A. incurvata* fell within the range of *A. kansuensis* and *A. yangii*, exhibiting an intermediate state between the two species. Additionally, these type specimens showed no significant differences in most floral quantitative traits when compared to *A. kansuensis*, *A. yangii* and *A. ecalcarata*, supporting the hypothesis that the type specimens of *A. incurvata* represent hybrid individuals. The continuous floral morphological variation likely reflects the manifestation of genetic admixture in floral traits.

Results of molecular and morphological analyses did not cluster samples from the populations SN61, SN65, SN66 and SN67 together with the type specimens of *A. incurvata*, but separated them into two groups. Group 1 (SN61 and SN65) was genetically and morphologically identified as *A. yabeana*. Nuclear genome SNPs analysis showed genetic composition identical to *A. yabeana*, while the 3D-PCA plot of floral traits clustered these populations together, with dehiscent anthers consistently yellow. Group 2 (SN66 and SN67) was genetically and morphologically assigned to *A. kansuensis*, exhibiting genetic similarity to *A. kansuensis* in SNP analyses, clustering together in floral trait 3D-PCA and displaying black dehiscent anthers.

The main distribution area of *A. incurvata* is the Qinling Mountains and Bashan Mountains, which constitute a biodiversity hotspot (Myers et al. 2000). The Qinba Mountains served as refugia for numerous species during the Quaternary glacial period and functioned as corridors for species migration and convergence (Hoorn et al. 2013; Qiu et al. 2011; Zhou et al. 2010), and different species frequently come into contact here, facilitating hybrid events and generating numerous hybrid offspring with admixed genetic backgrounds. It is well known that postzygotic reproductive isolation among species of *Aquilegia* is very weak (Prażmo 1961; Groh and Cronk 2020). *Aquilegia ecalcarata* and *A. chrysantha* Gray, which diverged long ago and are distributed in Asia and North America, can be artificially crossed (Prażmo 1961). In our study, *A. kansuensis* and *A. yangii* co-occur in the Qinba Mountains, enabling hybridization and backcrossing in their contact zones. Meanwhile, *A. ecalcarata* is also found in the Qinba Mountains, further facilitating interspecific hybridization. Furthermore, pollination studies of *Aquilegia* in the Qinba Mountains found that bumblebees serve as pollinators for spurred species (*A. incurvata* and *A. yabeana*) (Tang et al. 2007), suggesting that spurred species (*A. kansuensis* and *A. yangii*) in this region likely share similar pollinators. Thus, we hypothesize that the combined factors of weak postzygotic reproductive isolation, potentially shared pollinators and sympatric distribution promote extensive hybridization among *A. kansuensis*, *A. yangii* and *A. ecalcarata* in the Qinba Mountains, giving rise to numerous transitional forms. The holotype and paratypes of *A. incurvata* likely represent such a hybrid example, being similar to the natural populations exhibiting admixed genetic compositions of *A. kansuensis*, *A. yangii* and *A. ecalcarata* (Geng et al. 2024; Liu et al. 2025; Peng et al. 2025; Xue et al. 2021).

In summary, multiple pieces of evidence from this study show that these morphological traits, once used as diagnostic criteria, are not exclusive to *A. incurvata*; instead, they represent phenotypic reflections of introgression between three sympatric congeneric species—*A. kansuensis*, *A. yangii* and *A. ecalcarata*. This finding reveals the key limitation of relying solely on single morphological traits for species delimitation in taxa with frequent hybridization, such as the *Aquilegia*: the intermediate morphologies previously regarded as “unique to new species” may actually be the result of continuous gene flow among existing species (Sheidai et al. 2018; Chang et al. 2022; Webster et al. 2025).

For taxonomic practice, this work suggests that when describing new species within *Aquilegia* or other taxa with weak postzygotic reproductive isolation (Prażmo 1961; Groh and Cronk 2020), a comprehensive delimitation approach should be adopted by integrating phylogenetic relationships, population-level sampling data and morphological traits (Liu 2016; Hong 2020). This integrative taxonomic method not only avoids the issue of “taxonomic inflation” caused by misidentifying hybrid individuals as new species but also ensures that taxonomic treatment results are consistent with the evolutionary nature of species boundaries (Young et al. 2019; Mathews et al. 2025). Thereby, it guarantees that conservation efforts (e.g., priority conservation of endangered species) are not misdirected toward transient hybrid taxa.

## Additional information

### Use of AI

No use of AI was reported.

### Author ORCIDs

Ju-Qing Kang https://orcid.org/0000-0002-6421-0854

### Data availability

Raw genome sequence data can be accessed at the China National Center for Bioinformation (PRJCA041485).

## Supplementary materials

Supplementary material 1 Supplementary tables and figures Data type doc Explanation note **table SS1**. The morphology description in “Flora of China” of *A. incurvata* and its relatives. **table SS2**. Status of qualitative characteristic and its attribute. **figure S1**. The holotype and eight paratypes of *A. incurvata*. **figure S2**. Phylogenetic relationships of *Aquilegia* based on the multi-locus cpDNA dataset. **figure S3**. Phylogenetic relationships of *A. incurvata* and its relatives based on a multi-locus cpDNA dataset. **figure S4**. Cross-validation plot of admixture analysis. https://binary.pensoft.net/file/1626935This dataset is made available under the Open Database License (http://opendatacommons.org/licenses/odbl/1.0/). The Open Database License (ODbL) is a license agreement intended to allow users to freely share, modify, and use this Dataset while maintaining this same freedom for others, provided that the original source and author(s) are credited. Fei Gao, Jiao-Jie Li, Cheng Xue, Fang-Dong Geng, Lei Huang, Jian-Qiang Zhang, Xiao-Hui Zhang, Yi Ren, Ju-Qing Kang

## References

[B1] Alexander DH, Novembre J, Lange K (2009) Fast model-based estimation of ancestry in unrelated individuals. Genome Research 19: 1655–1664. 10.1101/gr.094052.109 PMC2752134 19648217

[B2] Anisimova M, Gil M, Dufayard JF, Dessimoz C, Gascuel O (2011) Survey of branch support methods demonstrates accuracy, power, and robustness of fast likelihood-based approximation schemes. Systematic Biology 60: 685–699. 10.1093/sysbio/syr041 PMC3158332 21540409

[B3] Chang JT, Chao CT, Nakamura K, Liu HL, Luo MX, Liao PC (2022) Divergence with gene flow and contrasting population size blur the species boundary in *Cycas* sect. Asiorientales, as inferred from morphology and RAD-seq data. Frontiers in Plant Science 13: 824158. 10.3389/fpls.2022.824158 PMC9125193 35615129

[B4] Erst AS, Luferov AN, Kuznetzov AA, Shmakov AI (2014) On the taxonomical status of *Aquilegia kansuensis* (Ranunculaceae). Turczaninowia 17: 24–25.

[B5] Fior S, Li M, Oxelman B, Viola R, Hodges SA, Ometto L, Varotto C (2013) Spatiotemporal reconstruction of the *Aquilegia* rapid radiation through next-generation sequencing of rapidly evolving cp DNA regions. New Phytologist 198: 579–592. 10.1111/nph.12163 23379348

[B6] Geng FD, Xie JH, Xue C, Sun L, Li JJ, Niu CY, Huang L, Zhang XH, Kang JQ, Kong HZ, Ren Y, Zhang JQ (2022) Loss of innovative traits underlies multiple origins of *Aquilegia ecalcarata*. Journal of Systematics and Evolution 60: 1291–1302. 10.1111/jse.12808

[B7] Geng FD, Liu MQ, Zhang XD, Wang LZ, Lei MF (2024) Genomics of hybrid parallel origin in *Aquilegia ecalcarata*. BMC Ecology and Evolution 24: 75. 10.1186/s12862-024-02266-7 PMC11155106 38844857

[B8] Grant V (1952) Isolation and hybridization between *Aquilegia formosa* and *A. pubescens*. Aliso: A Journal of Systematic and Floristic Botany 2: 341–360.

[B9] Groh JS, Cronk QC (2020) Phenotypic evidence for an extensive mosaic hybrid zone between two species of columbine. *Aquilegia flavescens* and *A. formosa*. Botany 98: 459–467. 10.1139/cjb-2020-0015

[B10] Guindon S, Dufayard JF, Lefort V, Anisimova M, Hordijk W, Gascuel O (2010) New algorithms and methods to estimate maximum-likelihood phylogenies: assessing the performance of PhyML 3.0. Systematic Biology 59: 307–321. 10.1093/sysbio/syq010 20525638

[B11] Hahn C, Bachmann L, Chevreux B (2013) Reconstructing mitochondrial genomes directly from genomic next-generation sequencing reads - a baiting and iterative mapping approach. Nucleic Acids Research 41: e129–e129. 10.1093/nar/gkt371 PMC3711436 23661685

[B12] Huang L, Geng FD, Fan JJ, Zhai W, Xue C, Zhang XH, Ren Y, Kang JQ (2022) Evidence for two types of *Aquilegia ecalcarata* and its implications for adaptation to new environments. Plant Diversity 44: 153–162. 10.1016/j.pld.2021.06.006 PMC9043306 35505982

[B13] Hong DY (2016) Biodiversity pursuits need a scientific and operative species concept. Biodiversity Science 24: 979–999.

[B14] Hong DY (2020) Gen-morph species concept - A new and integrative species concept for outbreeding organisms. Journal of Systematics and Evolution 58: 725–742. 10.1111/jse.12660

[B15] Hoorn C, Mosbrugger V, Mulch A, Antonelli A (2013) Biodiversity from mountain building. Nature Geoscience 6: 154. 10.1038/ngeo1742

[B16] Instituto Botanico Boreali-Occidentali (1974) *Aquilegia*. In: Instituto Botanico Boreali-Occidentali, Academiae Sinicae, (Eds) Flora Tsinlingensis 2. Beijing: Science Press, 234–236.

[B17] Jeffrey SG, Diana MP, Curtis RB, Quentin CBC (2018) On the origin of orphan hybrids between *Aquilegia formosa* and *Aquilegia flavescens*. AoB PLANTS 11(1): ply071. 10.1093/aobpla/ply071 PMC6341775 30687492

[B18] Kalyaanamoorthy S, Minh BQ, Wong TK, von Haeseler A, Jermiin LS (2017) ModelFinder: fast model selection for accurate phylogenetic estimates. Nature Methods 14: 587–589. 10.1038/nmeth.4285 PMC5453245 28481363

[B19] Katoh K, Standley DM (2013) MAFFT Multiple Sequence Alignment Software Version 7: Improvements in Performance and Usability. Molecular Biology and Evolution 30: 772–780. 10.1093/molbev/mst010 PMC3603318 23329690

[B20] Li H, Durbin R (2009) Fast and accurate short read alignment with Burrows-Wheeler transform. Bioinformatics 25: 1754–1760. 10.1093/bioinformatics/btp324 PMC2705234 19451168

[B21] Li H (2011) A statistical framework for SNP calling, mutation discovery, association mapping and population genetical parameter estimation from sequencing data. Bioinformatics 27: 2987–2993. 10.1093/bioinformatics/btr509 PMC3198575 21903627

[B22] Liu JQ (2016) The integrative species concept” and “species on the speciation way. Biodiversity Science 24: 1004–1008. 10.17520/biods.2016222

[B23] Liu HJ, Han BC, Mou HL, Xiao Y, Jiang YC, Kong HZ, Xu GX (2025) Unravelling the extensive phylogenetic discordance and evolutionary history of spurless taxa within the *Aquilegia ecalcarata* complex. New Phytologist 246: 1333–1349. 10.1111/nph.70039 40051377

[B24] Luo Y, Erst AS, Yang CX, Deng JP, Li L (2018) *Aquilegia yangii* (Ranunculaceae), a new species from western China. Phytotaxa 348: 289–296. 10.11646/phytotaxa.348.4.5

[B25] Mathews KG, Wheeler B, Silveira L (2025) Using integrative taxonomy to clarify species boundaries in Diervilla (bush-honeysuckle, Caprifoliaceae). Botanical Journal of the Linnean Society 208: 29–42. http://doi.10.1093/botlinnean/boae058

[B26] Myers N, Mittermeier RA, Mittermeier CG, da Fonseca GAB, Kent J (2000) Biodiversity hotspots for conservation priorities. Nature 403: 853–858. http://doi.10.1038/35002501 10.1038/35002501 10706275

[B27] Naciri Y, Linder HP (2015) Species delimitation and relationships: the dance of the seven veils. Taxon 64: 3–16. 10.12705/641.24

[B28] Nguyen LT, Schmidt HA, Von HA, Minh BQ (2015) IQ-TREE: a fast and effective stochastic algorithm for estimating maximum-likelihood phylogenies. Molecular Biology and Evolution 32: 268–274. 10.1093/molbev/msu300 PMC4271533 25371430

[B29] Niu Y-T, Jabbour F, Barrett RL, Ye J-F, Zhang Z-Z, Lu K-Q, Lu L-M, Chen Z-D (2018) Combining complete chloroplast genome sequences with target loci data and morphology to resolve species limits in *Triplostegia* (Caprifoliaceae). Molecular Phylogenetics and Evolution 129: 15–26. 10.1016/j.ympev.2018.07.013 30026123

[B30] Peng JC, He Z, Zhang ZQ (2025) Standing genetic variation and introgression shape the cryptic radiation of *Aquilegia* in the mountains of Southwest China. Communications Biology 8: 684. 10.1038/s42003-025-08120-w PMC12043930 40307563

[B31] Prażmo W (1961) Genetic studies on the genus *Aquilegia* L. II. Crosses between *Aquilegia ecalcarata* Maxim and *Aquilegia chrysantha* Gray. Acta Societatis Botanicorum Poloniae 30: 423–442.

[B32] Purcell S, Neale B, Todd-Brown K, Thomas L, Ferreira MA, Bender D, Maller J, Sklar P, Bakker PId, Daly MJ, Sham PC (2007) PLINK: A tool set for whole-genome association and population-based linkage analyses. American Journal of Human Genetics 81: 559–575. 10.1086/519795 PMC1950838 17701901

[B33] Qiu YX, Fu CX, Comes HP (2011) Plant molecular phylogeography in China and adjacent regions: Tracing the genetic imprints of Quaternary climate and environmental change in the world’s most diverse temperate flora. Molecular Phylogenetics and Evolution 59: 225–244. 10.1016/j.ympev.2011.01.012 21292014

[B34] Sheidai M, Teymoori H, Noormohammadi Z, Mehrabian AR, Koohdar F, Ghasemzadeh-Baraki S (2018) Species delimitation in the genus Tamarix: Morphological and molecular data. Phytotaxa 343: 101–115. 10.11646/phytotaxa.343.2.1

[B35] Tang LL, Yu Q, Sun JF, Huang SQ (2007) Floral traits and isolation of three sympatric *Aquilegia* species in the Qinling Mountains, China. Plant Systematics and Evolution 267: 121–128. 10.1007/s00606-007-0574-6

[B36] Wang B, Jiang J, Xie F, Li C (2012) Postglacial colonization of the Qinling Mountains: phylogeography of the swelled vent frog (*Feirana quadranus*). PLOS ONE 7: e41579. 10.1371/journal.pone.0041579 PMC3405020 22848532

[B37] Webster CP, Paris M, Olivares I, Wojciechowski MF, Kessler M, Sanín MJ (2025) Divergence and Selection in a Cryptic Species Complex (Geonoma undata: Arecaceae) in the Northern Andes of Colombia. Genome Biology and Evolution 17: evaf130. 10.1093/gbe/evaf130 PMC12279445 40691772

[B38] Xie J, Zhao H, Li K, Zhang R, Jiang Y, Wang M, Guo X, Yu B, Kong H, Jiao Y, Xu G (2020) A chromosome-scale reference genome of *Aquilegia oxysepala* var. kansuensis. Horticulture Research 7: 113. 10.1038/s41438-020-0328-y PMC7326910 32637141

[B39] Xue C, Geng F-D, Zhang X-Y, Chang X-P, Kang J-Q, Huang L, Zhang J-Q, Ren Y (2020) Morphological variation pattern of *Aquilegia ecalcarata* and its relatives. Journal of Systematics and Evolution 58: 221–233. 10.1111/jse.12494

[B40] Xue C, Geng FD, Li JJ, Zhang DQ, Gao F, Huang L, Zhang XH, Kang JQ, Zhang JQ, Ren Y (2021) Divergence in the *Aquilegia ecalcarata* complex is correlated with geography and climate oscillations: evidence from plastid genome data. Molecular Ecology. 10.1111/mec.16151 34448283

[B41] Xu X-X, Cheng F-Y, Peng L-P, Sun Y-Q, Hu X-G, Li S-Y, Xian H-L, Jia K-H, Abbott RJ, Mao J-F (2019) Late Pleistocene speciation of three closely related tree peonies endemic to the Qinling–Daba Mountains, a major glacial refugium in Central China. Ecology and Evolution 9: 7528–7548. 10.1002/ece3.5284 PMC6635923 31346420

[B42] Young MK, Smith RJ, Pilgrim KL, Fairchild MP, Schwartz MK (2019) Integrative taxonomy refutes a species hypothesis: The asymmetric hybrid origin of *Arsapnia arapahoe* (Plecoptera, Capniidae). Ecology and Evolution 9: 1364–1377. 10.1002/ece3.4852 PMC6374720 30805166

[B43] Zeng CX, Hollingsworth PM, Yang J, He ZS, Zhang ZR, Li DZ, Yang JB (2018) Genome skimming herbarium specimens for DNA barcoding and phylogenomics. Plant Methods 14: 43. 10.1186/s13007-018-0300-0 PMC5987614 29928291

[B44] Zhai W, Duan X, Zhang R, Guo C, Li L, Xu G, Shan H, Kong H, Ren Y (2019) Chloroplast genomic data provide new and robust insights into the phylogeny and evolution of the Ranunculaceae. Molecular Phylogenetics and Evolution 135: 12–21. 10.1016/j.ympev.2019.02.024 30826488

[B45] Zhang D, Gao F, Jakovlić I, Zou H, Zhang J, Li WX, Wang GT (2020) PhyloSuite: an integrated and scalable desktop platform for streamlined molecular sequence data management and evolutionary phylogenetics studies. Molecular Ecology Resources 20: 348–355. 10.1111/1755-0998.13096 31599058

[B46] Zhou T-H, Li S, Qian Z-Q, Su H-L, Huang Z-H, Guo Z-G, Dai P-F, Liu Z-L, Zhao G-F (2010) Strong phylogeographic pattern of cpDNA variation reveals multiple glacial refugia for *Saruma henryi* Oliv. (Aristolochiaceae), an endangered herb endemic to China. Molecular Phylogenetic Evolution 57: 176–188. 10.1016/j.ympev.2010.07.001 20637294

