# Supplementary material for: Combining nuclear genome, chloroplast fragments and morphology to solve the species delimitation of Aquilegia incurvata (Ranunculaceae)
Source: PhytoKeys. 2026 May 8;274:165–83. doi: 10.3897/phytokeys.274.161927 (PMC13179504; doi:10.3897/phytokeys.274.161927)
Supplement: Supplementary material 1 — Supplementary tables and figures [file phytokeys-274-165_article-161927__-s001.doc]

**Supporting information**

**Table S1.** The morphology description in *Flora of China* of *A. incurvata* and its relatives.

| Species | Sepal length × width (mm) | Petal blade length (mm) | Spur length (mm) | Stamen length (mm) | Follicles length (mm) | Colour of petal blade |
| --- | --- | --- | --- | --- | --- | --- |
| *A. incurvata* | **14-18**×？ | 7-8 | **12-15** | **5-9** | **14-15** | purple |
| *A. kansuensis* | **16-25**×8-12 | 10-13 | **15-20** | 15-20 | **12-17** | yellowish white |
| *A. yangii* ^a^ | **16-19**×？ | 11-14 | 9-11 | — | **18-28** | yellow |
| *A. yabeana* | **(16-) 20-26**×7-10 | 12-15 | **17-20** | -12 | **(12-) 15-20** | purple |
| *A. ecalcarata* | **10-14**×4-6 | 10-14 | 4-5 | **5-7** | 8-11 | purple |

In bold font: Descriptive characters of *A. incurvata* specimens that overlap with those of other closely related species.

a: data from Luo et al. (2018).

**Table S2.** Status of qualitative characteristic and its attribute.

| **characteristics** | **characteristic state** |
| --- | --- |
| Colour of dehiscent anthers | 0, black; 1, yellow |

**Figure S1.** The holotype and eight paratypes of *A. incurvata.*

***
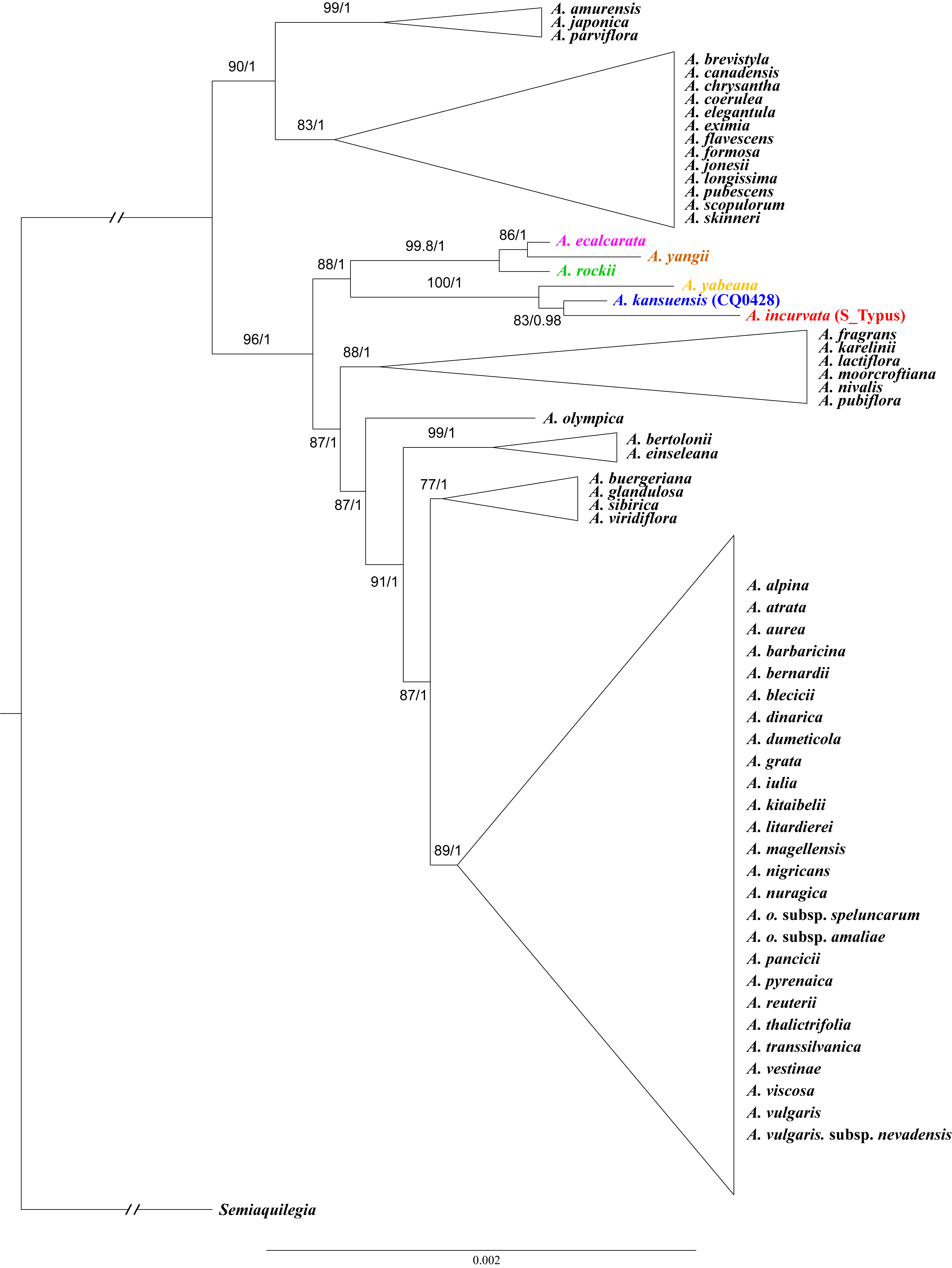
***

**Figure S2.** Phylogenetic relationships of *Aquilegia* based on the multi-locus cpDNA dataset. The branch numbers represent ML bootstrap values / aBayes values. The individual of *A. kansuensis* in Fior’s research (Fior et al*.* 2013) was collected from Gansu, which was revised to a new species - *A. yangii* (Luo et al*.* 2018). Therefore, we added an individual of *A. kansuensis* (CQ0428) for analysis.


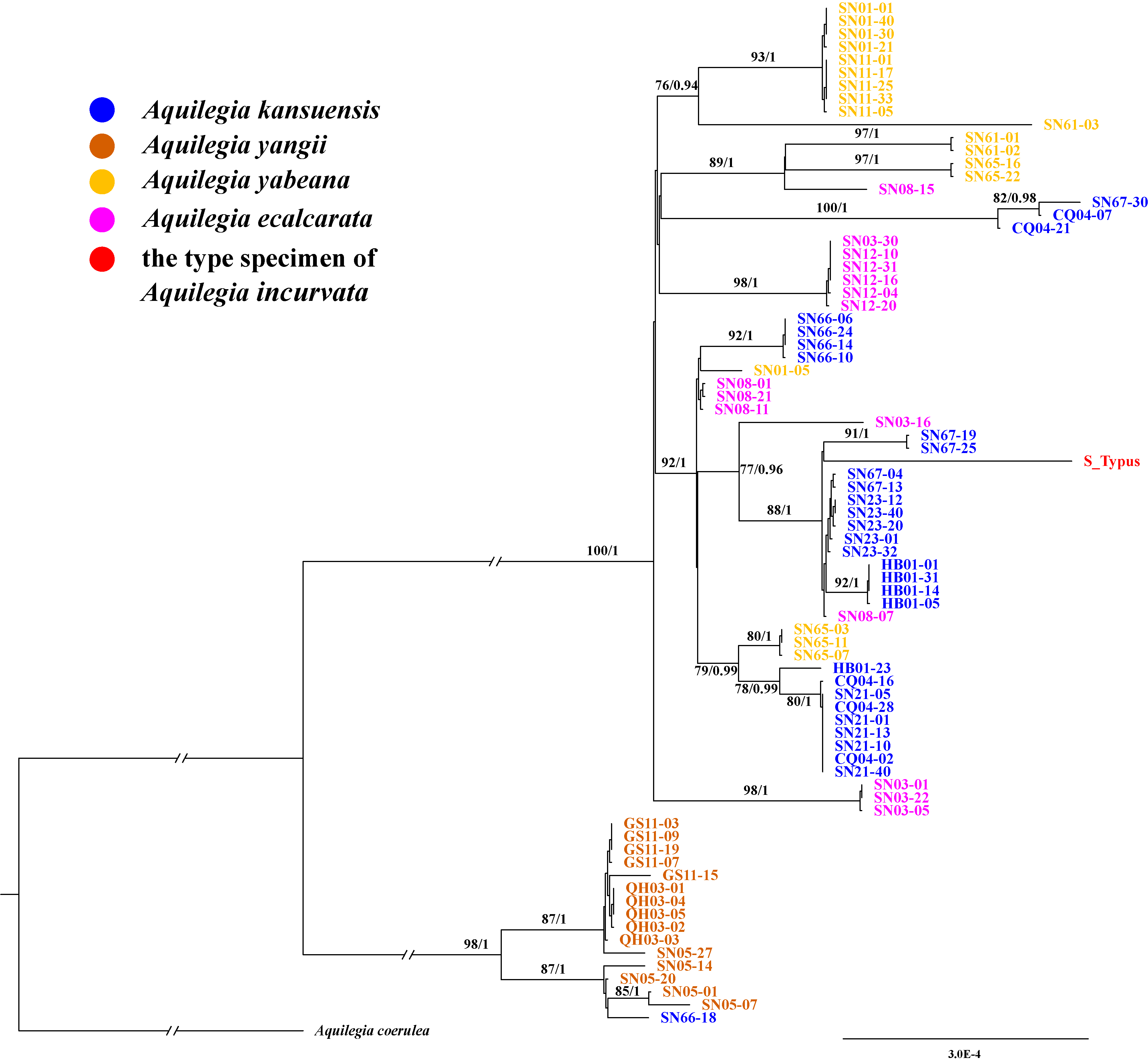


**Figure S3**. Phylogenetic relationships of *A. incurvata* and its relatives based on a multi-locus cpDNA dataset. The branch numbers represent ML bootstrap values / aBayes values.

**
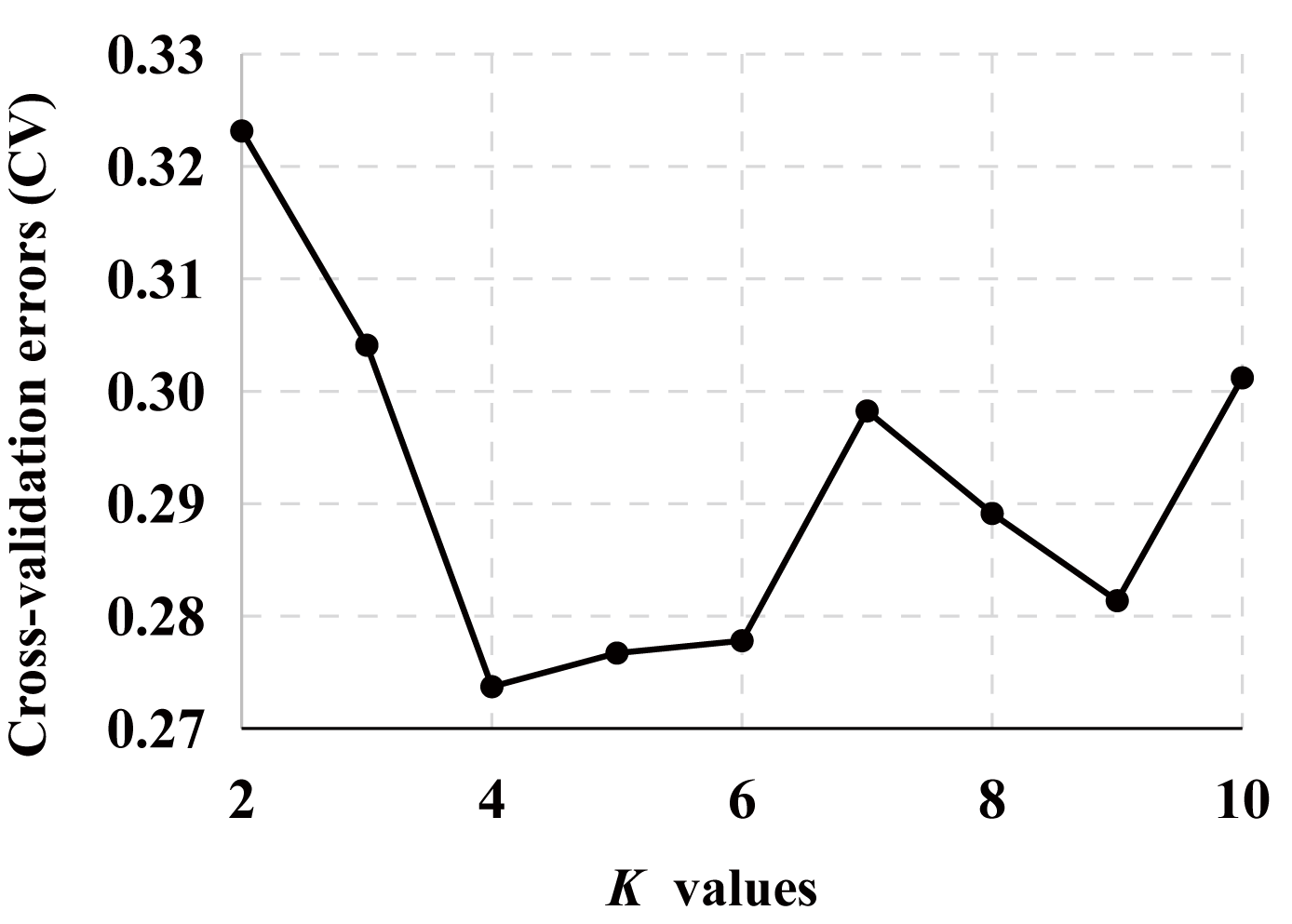
**

**Figure S4.** Cross-validation plot of admixture analysis. Admixture with cross-validation for *K* values 2-10.
